# Complex Management of Extensive Stanford Type B Aortic Dissection With Multiorgan Failure and Acute Limb Ischemia Treated by Femoro-Femoral Bypass and Complicated by Iatrogenic Brachial Arteriovenous Fistula: A Case Report

**DOI:** 10.7759/cureus.88173

**Published:** 2025-07-17

**Authors:** Maciej Mach, Tomasz Ostrowski, Aleksandra Giba, Natalia Sudol, Przemyslaw Kabala, Wawrzyniec Jakuczun, Rafał Maciąg, Michał Sajdek, Zbigniew Gałązka

**Affiliations:** 1 Department of General, Vascular, Endocrine, and Transplant Surgery, Medical University of Warsaw, Warsaw, POL; 2 Department of Vascular Surgery, Samodzielny Publiczny Specjalistyczny Szpital Zachodni im. św. Jana Pawła II, Grodzisk Mazowiecki, POL; 3 2nd Department of Clinical Radiology, Medical University of Warsaw, Warsaw, POL

**Keywords:** acute lower limb ischemia, arteriovenous fistula failure, femoro-femoral bypass graft, iatrogenic complication, type b acute aortic dissection

## Abstract

We present the case of a 62-year-old woman with a history of long-standing, poorly controlled arterial hypertension, with average home blood pressure readings of approximately 140-160/100-110 mmHg despite treatment with ramipril, who was urgently admitted due to acute critical ischemia of the left lower limb. Imaging revealed an extensive Stanford type B aortic dissection (TBAD) involving the thoracoabdominal aorta and iliac arteries, with significantly impaired perfusion of the left leg. Due to the severity of limb ischemia and limitations to immediate endovascular repair, an urgent right-to-left femoro-femoral bypass was performed as a limb-saving procedure. Postoperatively, the patient developed multiorgan complications, including acute kidney injury (AKI) requiring hemodialysis and bowel ischemia, which were managed conservatively. The definitive repair of the dissection was achieved through thoracic endovascular aortic repair (TEVAR). Several weeks later, the patient presented with a gradually enlarging, painless, pulsatile mass in the right antecubital fossa, corresponding to a prior arterial access site. Clinical features, including a palpable thrill and audible bruit, were consistent with an iatrogenic arteriovenous fistula (AVF), which was surgically excised with the preservation of arterial flow and uneventful recovery. This case underscores the complex and life-threatening course of complicated type B aortic dissection with peripheral malperfusion, the necessity of staged hybrid management, and the importance of long-term follow-up for detecting delayed iatrogenic complications associated with endovascular procedures.

## Introduction

Acute limb ischemia (ALI), defined as a sudden decrease in limb perfusion threatening viability within 14 days, is most commonly caused by embolism or thrombosis, often due to peripheral arterial disease (PAD) or atrial fibrillation [1-3]. Clinical symptoms follow the "six Ps" (pain, pallor, pulselessness, paresthesia, poikilothermia, and paralysis) [1], with diagnosis based on physical examination and imaging [2,3]. ALI is classified by the Rutherford system to guide treatment, ranging from anticoagulation to urgent revascularization or amputation [1,2,4]. Endovascular methods, including catheter-directed thrombolysis and mechanical thrombectomy, are increasingly preferred due to their minimally invasive nature and efficacy [5,6]. Aortic dissection, another vascular emergency, results from a tear in the intimal layer of the aorta, forming a false lumen, with the Stanford classification guiding treatment: type A requires urgent surgery, while type B is often managed medically unless complications occur [7-9]. Hypertension is the most common risk factor, with connective tissue disorders and aortic anomalies contributing as well [10]. Presenting symptoms vary but typically include tearing chest or back pain and possible malperfusion signs such as limb ischemia or neurological deficiency [11,12]. Rapid blood pressure control, pain management, and early diagnosis are crucial, with endovascular techniques used in selected type B cases [9,13]. Arteriovenous fistula (AVF) is an abnormal connection between arteries and veins that may form iatrogenically after vascular procedures. Diagnosis is based on duplex ultrasound, supported by computed tomography (CT) angiography to assess flow dynamics and anatomy [14,15]. While many AVFs close spontaneously, persistent or symptomatic cases may require endovascular (e.g., coils and stent grafts) or surgical (e.g., ligation and excision) treatment, with the main goal being the closure of the fistula while maintaining distal perfusion [16,17].

We present a 62-year-old woman with long-standing, poorly controlled hypertension with average readings of 140-160/100-110 mmHg, who was urgently hospitalized due to acute critical ischemia of the left lower limb secondary to extensive Stanford type B aortic dissection (TBAD), complicated by multiorgan failure, requiring staged surgical and endovascular interventions including femoro-femoral bypass, thoracic endovascular aortic repair (TEVAR), and the subsequent management of reperfusion injury, bowel ischemia, and an iatrogenic brachial arteriovenous fistula.

## Case presentation

A 62-year-old woman with a history of poorly controlled, long-standing arterial hypertension (average home blood pressure readings of approximately 140-160/100-110 mmHg), chronically treated with ramipril 10 mg daily, was admitted overnight to the vascular surgery department of a local hospital on an emergency basis due to symptoms of acute critical ischemia of the left lower limb, posing an immediate threat to limb viability. Symptoms began abruptly in the late morning hours, initially manifesting as generalized weakness and chest discomfort, followed by the onset of lumbar pain and progressively worsening paresis of the left lower extremity, accompanied by severe acute pain. No additional significant comorbidities were identified in the medical history. On admission, the patient was alert and oriented, with preserved cognitive function. She was hemodynamically and respiratorily stable, with a blood pressure of 200/98 mmHg and a regular heart rate of 90 beats per minute (bpm). The following are the laboratories done in the hospital after admission (Table 1). Physical examination showed that the entire left lower limb was notably colder to the touch, with a distinct cyanotic discoloration between the upper and middle part of the thigh. No active movement was observed in the left foot or toes, and only minimal movement was present at the left knee. Profound sensory deficits were noted in the left lower leg and foot.

**Table 1:** Laboratory Test Results on Emergency Department Admission

Test	Example Result	Reference Range	Clinical Significance
C-reactive protein (CRP)	145 mg/L	<5 mg/L	Strong inflammatory response due to ischemia and tissue necrosis
White blood cell (WBC) count	18.7 × 10⁹/L	4.0-10.0 × 10⁹/L	Suggests acute inflammation or systemic stress
Hemoglobin (Hb)	11.2 g/dL	12-16 g/dL	Mild anemia, possibly due to inflammation or early hemolysis
Platelet count	360 × 10⁹/L	150-400 × 10⁹/L	Normal or reactive thrombocytosis
Creatine kinase (CK)	14,387 U/L	26-192 U/L	Suggests rhabdomyolysis due to prolonged muscle ischemia
Myoglobin	11,283 ng/mL	<72 ng/mL	Indicates extensive muscle injury
Lactate	5.2 mmol/L	0.5-2.2 mmol/L	Indicates tissue hypoperfusion and anaerobic metabolism
Creatinine	2.05 mg/dL	0.5-1.1 mg/dL	Suggests acute kidney injury (possibly from myoglobinuria)
Blood urea nitrogen (BUN)	62 mg/dL	15-48 mg/dL	May reflect impaired renal perfusion or catabolism
Estimated glomerular filtration rate (eGFR)	29 mL/minute	>60 mL/minute	Decreased kidney function
Potassium (K⁺)	5.8 mmol/L	3.6-5.0 mmol/L	Risk of arrhythmia from cell breakdown
Sodium (Na⁺)	137 mmol/L	137-145 mmol/L	Within normal range
Glucose	118 mg/dL	70-99 mg/dL	Mild stress hyperglycemia
Troponin I	52 ng/L	<15 ng/L	Possible myocardial strain or secondary ischemia
D-dimer	1,200 ng/mL	<500 ng/mL	Nonspecific marker of thrombosis or embolism
Fibrinogen	685 mg/dL	200-400 mg/dL	Elevated in acute-phase reaction
Arterial blood gas (ABG)	pH, 7.28; HCO₃⁻, 17 mmol/L; lactate, 5.2 mmol/L	pH: 7.35-7.45	Metabolic acidosis with lactic acidemia
Prothrombin time (PT)	13.4 seconds	11-13.5 seconds	Slightly prolonged, consider coagulation status
Activated partial thromboplastin time (aPTT)	34.2 seconds	25-36 seconds	Normal or mildly elevated
International normalized ratio (INR)	1.1	0.9-1.2	Within normal limits

**Figure 1:**
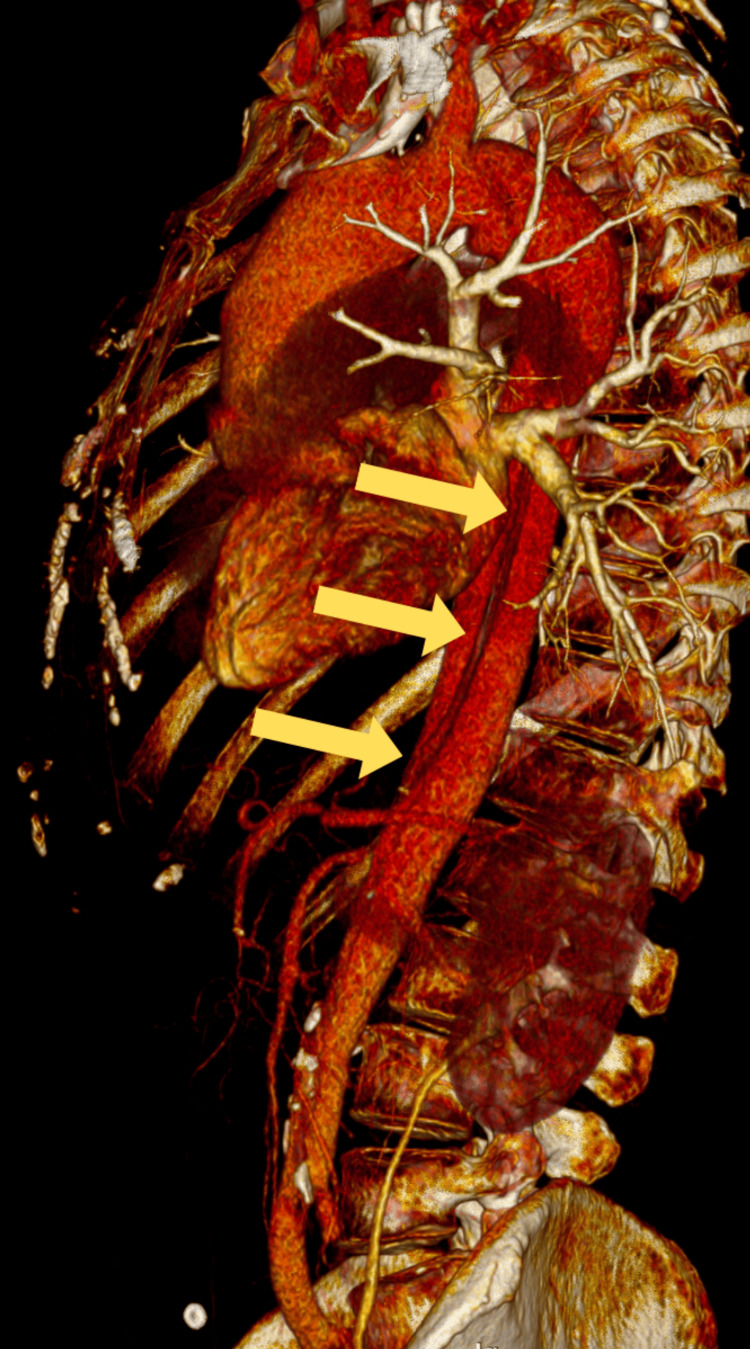
3D CT Angiographic Reconstruction The yellow arrow indicates aortic dissection 3D, three-dimensional; CT, computed tomography

Three-dimensional (3D) volume-rendered CT angiography of the abdominal aorta and lower extremities revealed significant asymmetry in perfusion between the right and left lower limbs, consistent with acute-on-chronic ischemia of the left side (Figure 1). The reconstruction demonstrated robust arterial enhancement of the right lower extremity, with uninterrupted opacification of the common femoral artery (CFA), superficial femoral artery (SFA), popliteal artery, and infrapopliteal artery down to the pedal vessels, indicating preserved perfusion and patency throughout the arterial tree.

**Figure 2:**
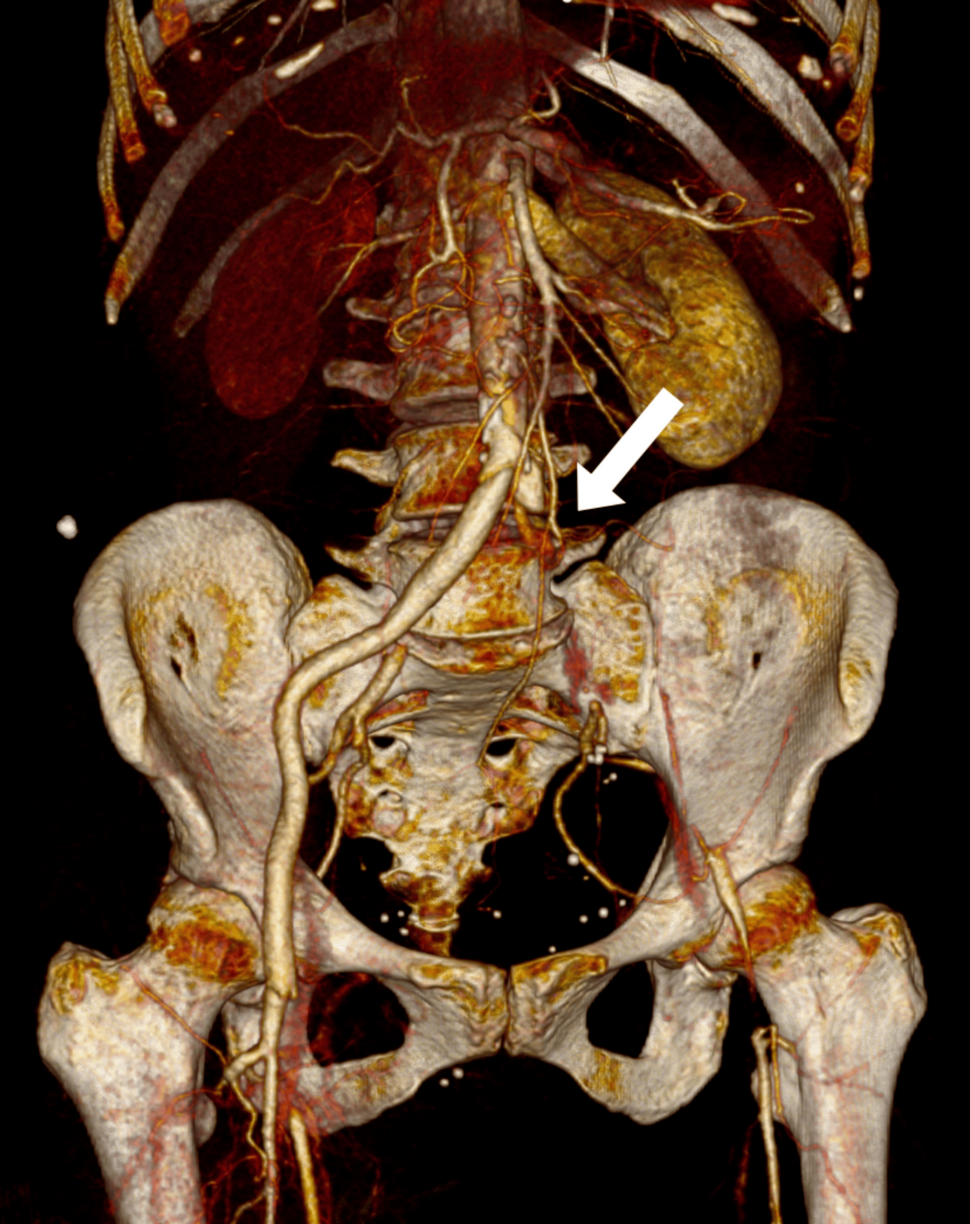
3D CT Angiographic Reconstruction The white arrow indicates a critical absence of flow in the left common iliac artery 3D, three-dimensional; CT, computed tomography

In contrast, the left lower limb showed evidence of critical ischemia (Figure 2). The SFA, popliteal artery, and all three tibial vessels were either non-opacified or exhibited only faint, threadlike residual contrast signal, consistent with near-complete or complete occlusion. The CFA appeared hypoperfused, with only partial filling evident on reconstruction. These findings corresponded to acute critical ischemia of the left lower limb, correlating clinically with absent distal pulses and motor/sensory deficiency.

Surgical procedure

**Figure 3:**
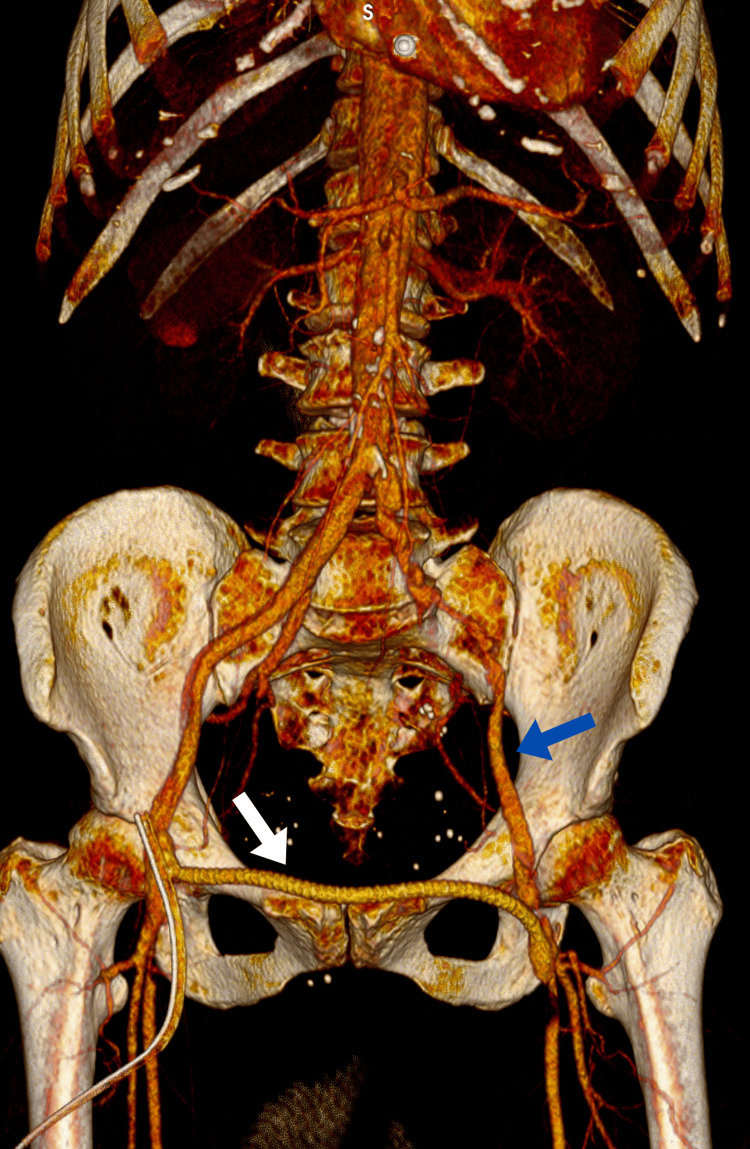
Postoperative 3D CT Angiographic Reconstruction The white arrow indicates a right-to-left femoro-femoral bypass graft. The blue arrow indicates restored perfusion in the left lower limb 3D, three-dimensional; CT, computed tomography

Due to the dissection of the aorta and iliac arteries and the good perfusion of the right leg, it was decided to perform a right-to-left femoro-femoral bypass graft. The placement of a stent graft was not feasible, so in order to prevent limb loss, a surgical bypass was performed. Bilateral longitudinal groin incisions were made. The CFAs and their bifurcations were carefully exposed bilaterally. On the right side, the femoral artery (FA) was soft and pulsatile. Following a longitudinal arteriotomy, a full-length dissection of the intimal layer was observed. The dissected intima was reattached to the vessel wall using interrupted 6-0 polypropylene sutures, and an end-to-side anastomosis was performed with a 6 mm reinforced polytetrafluoroethylene (PTFE) graft. The graft was passed through in an S-shape configuration above the pubic symphysis to reach the left groin (Figure 3). In the left groin, the complete dissection of the CFA and approximately 5 cm of the SFA was observed. Pulsation was weakly palpable only in the CFA. A longitudinal arteriotomy revealed the concentric separation of the arterial wall, forming a true and a false lumen, the outer being false and the inner true. The arteriotomy extended through both layers, which were then approximated with multiple interrupted 6-0 polypropylene sutures, reconstructing the arterial integrity. Retrograde backflow from the left profunda femoris artery (PFA) was robust and adequate from the SFA. The blood flow through the graft was pulsatile. A distal end-to-side anastomosis of the graft to the reconstructed left CFA was performed using a continuous 5-0 polypropylene suture. Upon the release of vascular clamps, both anastomoses were hemostatic, and blood flow was restored in the PFA, SFA, and circumflex femoral arteries bilaterally. Due to the persistent refilling of the false lumen on the left side, the decision was made to ligate the left CFA just proximal to the graft anastomosis. The hemodynamic effect was satisfactory. A hemostatic agent (Oxicel® Fibrillar, Betatech Medical, Istanbul, Turkey) was applied around both anastomotic sites. Bilateral 16F Redon drains were inserted. Multilayer wound closure was performed, followed by sterile dressings. Postoperatively, there was notable improvement in the perfusion of the left lower limb.

On the first postoperative day, clinical evaluation revealed improved perfusion of the left lower limb, with partial return of foot movements and minimal toe mobility. Sensory function in the left lower leg and foot also showed improvement. By morning, the limb was warm throughout. Due to signs of developing revascularization edema, the limb was elevated, although fasciotomy was not required. Persistent arterial hypertension was managed with an intravenous (IV) infusion of urapidil at 4 mg/hour via an infusion pump, successfully reducing blood pressure to 120/60 mmHg. The patient reported significant postoperative pain, for which a continuous infusion of oxycodone was initiated. Bilateral surgical drains showed only trace amounts of fluid. Postoperative diuresis was preserved, with a total urine output of 1,500 mL by morning. However, progressive dark discoloration of the urine was noted. On the third postoperative day, nephrology consultation was requested due to progressive laboratory signs of systemic inflammation and acute kidney injury (AKI) (Table 2), likely secondary to ischemia and reperfusion injury. The patient remained hemodynamically and respiratorily stable, with blood pressure of 148/60 mmHg and a regular heart rate of 90 bpm.

**Table 2:** Postoperative Laboratory Findings BUN, blood urea nitrogen; eGFR, estimated glomerular filtration rate

Test	Example Result	Reference Range
C-reactive protein (CRP)	220 mg/L	<5 mg/L
White blood cell (WBC) count	21.4 × 10⁹/L	4.0-10.0 × 10⁹/L
Creatinine	3.1 mg/dL	0.5-1.1 mg/dL
Urea (BUN)	74 mg/dL	15-48 mg/dL
eGFR	19 mL/minute	>60 mL/minute
Potassium (K⁺)	6.2 mmol/L	3.6-5.0 mmol/L
Myoglobin	12,462 ng/mL	<72 ng/mL
Creatine kinase (CK)	11,200 U/L	26-192 U/L
Lactate	3.6 mmol/L	0.5-2.2 mmol/L
Hemoglobin (Hb)	10.1 g/dL	12-16 g/dL
Urinalysis	Dark brown urine, positive for myoglobin	-
Aspartate aminotransferase (AST)	98 U/L	<40 U/L
Alanine aminotransferase (ALT)	52 U/L	<41 U/L

Postoperative treatment focused on the correction of anemia, maintenance of stable glycemia, adequate hydration targeting a daily urine output of 2,500-3,500 mL, and close monitoring of renal function, inflammatory markers, electrolytes, and fluid balance. Treatment was initiated with 1,500 mL of 0.9% NaCl intravenously, followed by two additional 500 mL infusions of 0.9% NaCl each containing 600 mg of N-acetylcysteine (ACC) as prophylaxis against contrast nephropathy. Oral hydration was also encouraged. To support diuresis, furosemide was administered at a dose of 60 mg IV three times daily. Empiric broad-spectrum antibiotic therapy included meropenem 500 mg IV twice daily, with the addition of linezolid 600 mg IV twice daily if indicated by blood culture results. Supportive medications included continued use of a proton pump inhibitor (PPI) and low-molecular-weight heparin, Clexane, for prophylaxis. At that time, renal replacement therapy was not required. A temporary central venous catheter was inserted into the right internal jugular vein, and the first session of hemodialysis (duration: 2-5 hours) with ultrafiltration of 500 mL was performed. The patient maintained preserved residual diuresis. Hemodynamic status remained stable throughout the procedure. At the beginning of dialysis, arterial blood pressure was elevated at 180/77 mmHg; sublingual captopril 12.5 mg was administered. Subsequently, blood pressure remained at 175/76 mmHg, prompting the administration of sublingual nitrendipine 20 mg. Oxygen therapy was continued via nasal cannula at 3 L/minute, with oxygen saturation maintained at 97%. Due to the patient's deteriorating condition and the lack of available treatment options for the aortic dissection at the current facility, a decision was made to transfer her to a higher-level referral center.

**Figure 4:**
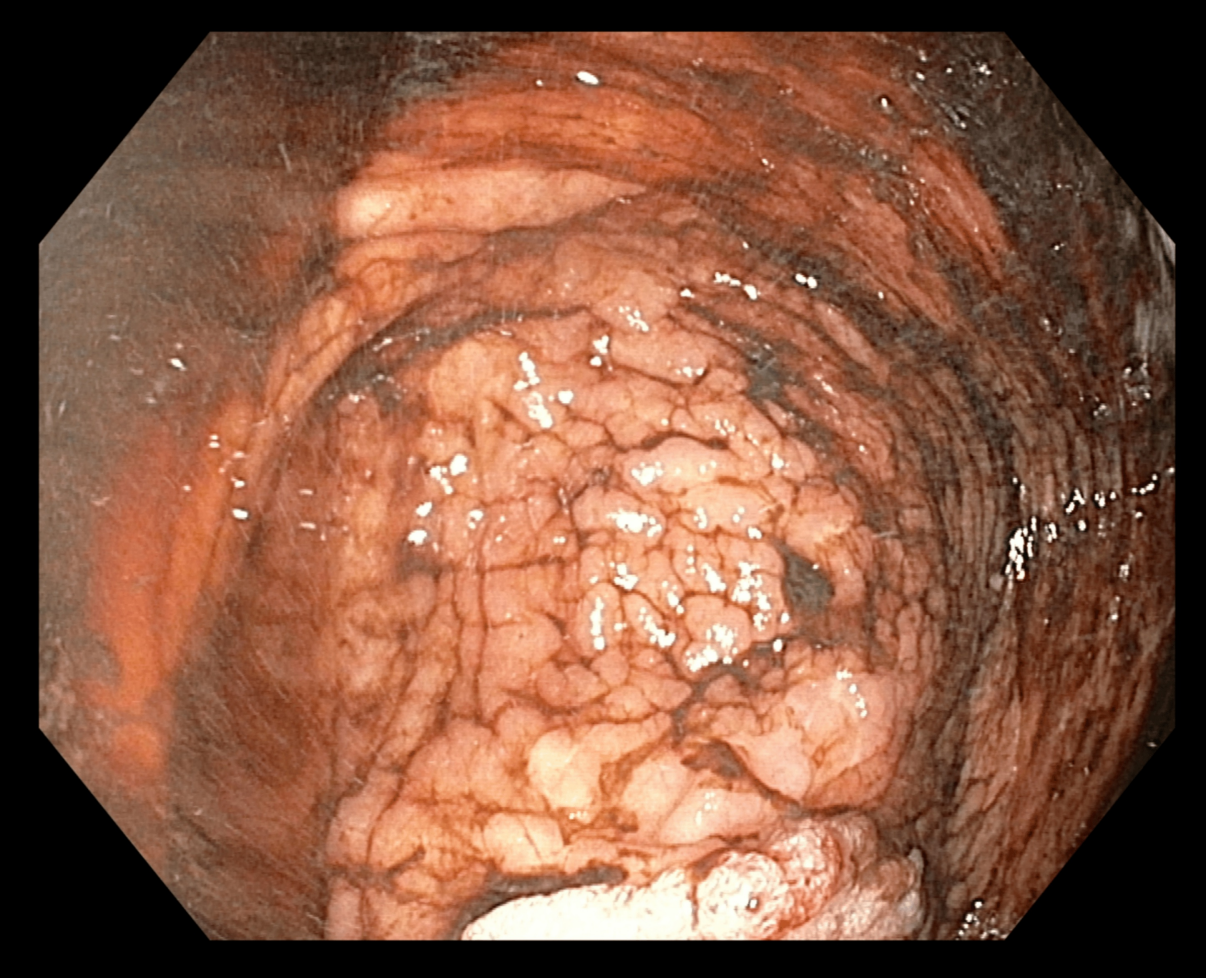
Sigmoidoscopy: Ischemic Changes and Ulcerations in the Colon

The patient was admitted to our department a few days later for the corrective treatment of an acute type B thoracoabdominal aortic dissection using a stent graft, complicated by multiorgan failure, ischemia of the left lower limb, and bowel ischemia. On admission, the patient's condition was assessed as moderately severe. The patient reported abdominal pain and diffuse chest discomfort, accompanied by intermittent episodes of belching without gastric content. Vital signs revealed a heart rate of 96 bpm and a blood pressure of 133/57 mmHg. Oxygen saturation was 95% on supplemental oxygen delivered via a high-flow system. Pulmonary auscultation revealed normal vesicular breath sounds, slightly diminished at the base of the right lung. The examination of the left lower limb demonstrated swelling, impaired mobility, and reduced sensation. Electrocardiogram showed a sinus rhythm at 100 bpm, with no evidence of acute myocardial infarction. Sigmoidoscopy revealed findings suggestive of ischemic changes; in the terminal ileum, extensive, progressive bleeding ulcers were observed; Bauhin's valve appeared edematous and ulcerated as well. The entire colon was filled with bloody content originating from the small intestine​​​​​​ (Figure 4).

**Figure 5:**
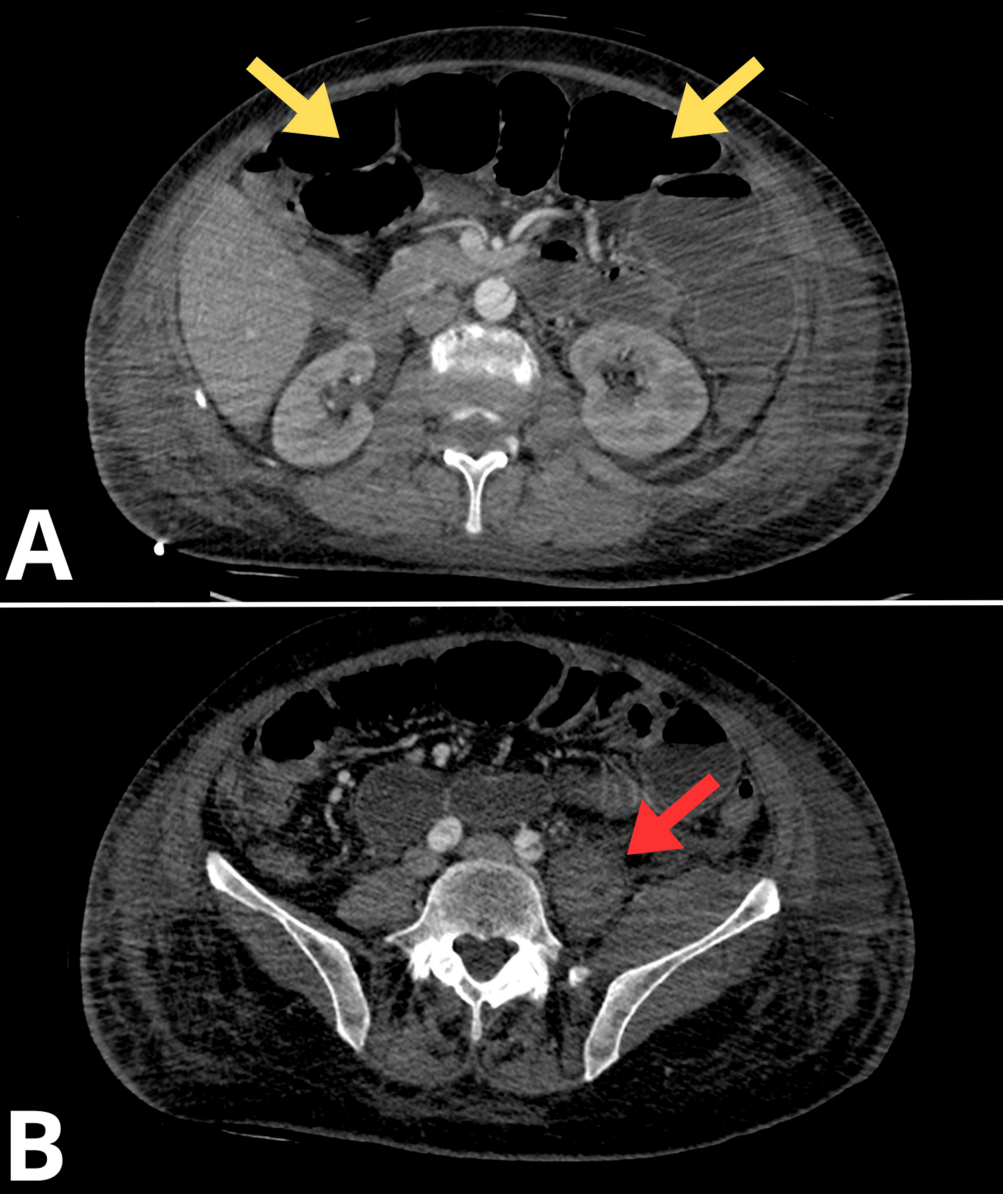
Angio-CT Transverse Scan of the Abdomen (A) Yellow arrows indicate dilated intestinal loops. (B) The red arrow indicates the swollen left iliopsoas muscle CT: computed tomography

Angio-CT revealed markedly dilated small bowel loops (up to 58 mm) with fluid levels, consistent with bowel obstruction, and a small volume of hyperdense fluid in the pelvic cavity (Figure 5A). A left-sided pleural effusion (~37 mm) was present, accompanied by adjacent atelectatic or inflammatory changes. There was a thickening of the left iliopsoas muscle, raising suspicion for a hematoma measuring approximately 29 × 61 mm (Figure 5B).

**Figure 6:**
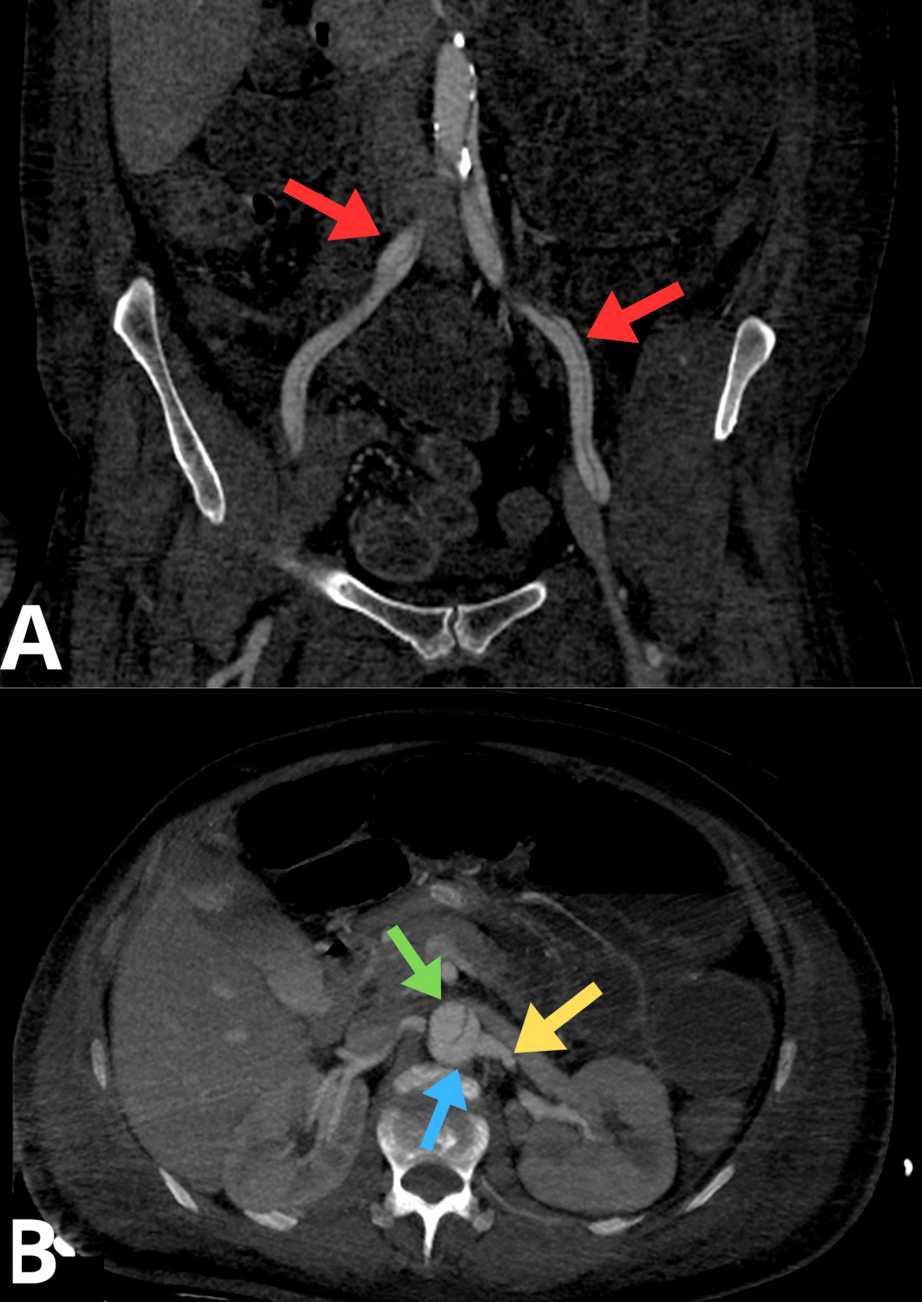
Angio-CT Scans: (A) Coronal Plane and (B) Transverse Plane The red arrow indicates dissection in both common iliac arteries. The green arrow indicates the true lumen. The blue arrow indicates the false lumen. The yellow arrow indicates the left renal artery originating from the false lumen CT: computed tomography

A Stanford type B aortic dissection was visualized, beginning just distal to the left subclavian artery. At the thoracoabdominal transition near the diaphragmatic hiatus, the true lumen appeared severely narrowed and located anteriorly. The dissection extended into both common iliac arteries (CIAs) (Figure 6A). The celiac trunk, superior mesenteric artery (SMA), right renal artery, and inferior mesenteric artery originated from the true lumen, while the left renal artery arose from the false lumen, which was contrast-filled (Figure 6B). The left CIA was significantly narrowed, with mural hypodense lesions; the left internal iliac artery (IIA) was non-opacified along a long segment, and the distal left external iliac artery (EIA) was severely stenosed, likely due to thrombi or partially thrombosed dissection channels. The segmental narrowing of the left femoral vein was also observed. On the right, the CIA was dissected with both lumens contrast-opacified, and the right internal, external, and common femoral arteries remained patent.

Surgical procedure

**Figure 7:**
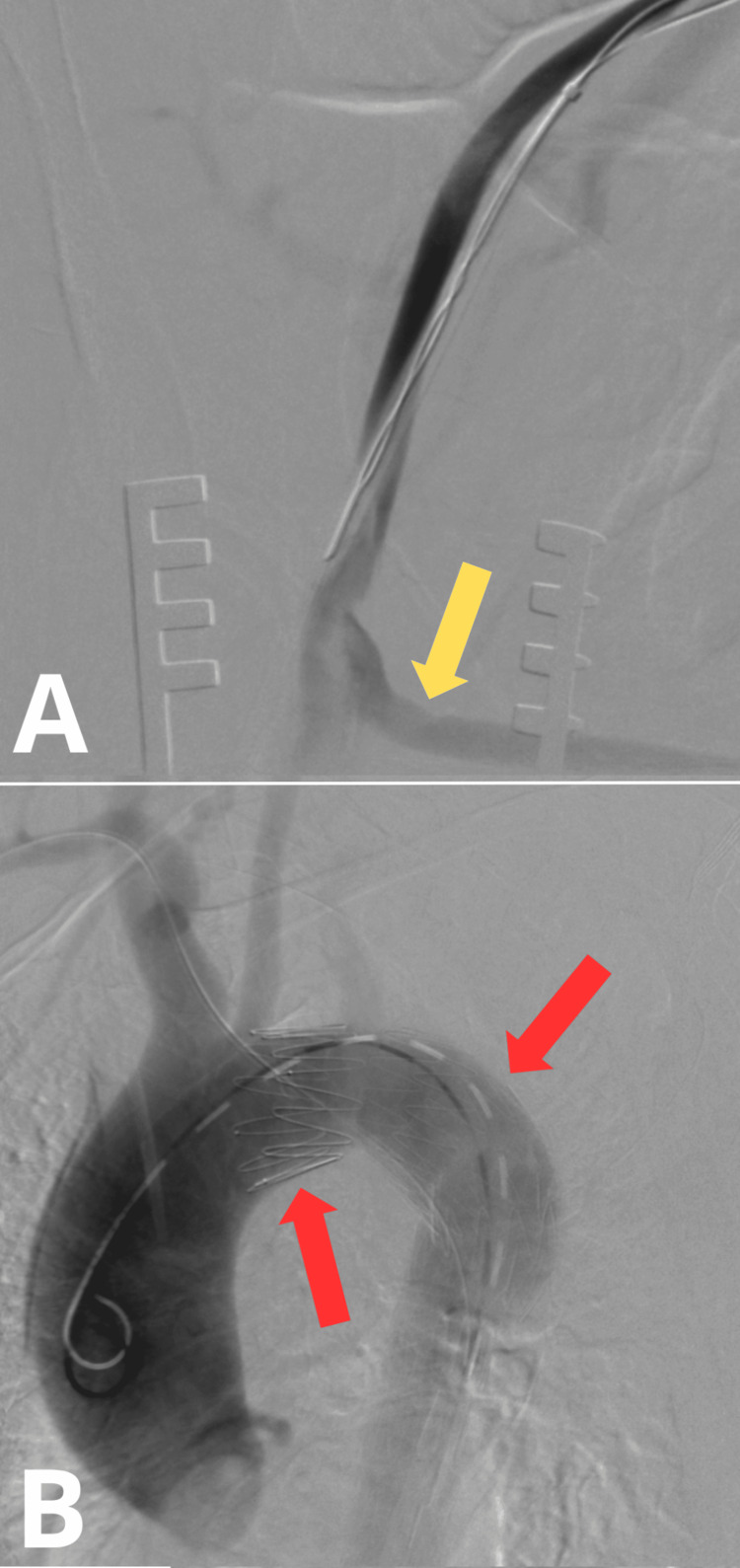
Intraoperative angio-CT Scan: (A) the View of the Patent Femoro-Femoral Bypass Graft and (B) the View of the Stent Graft in the Aortic Arch The yellow arrow indicates the left femoro-femoral bypass graft. The red arrow indicates the stent graft CT: computed tomography

On May 26, 2023, under general anesthesia, a repair procedure of an abdominal aortic aneurysm using a stent graft was performed. During the procedure, acute type B thoracoabdominal aortic dissection was confirmed, as well as a patent femoro-femoral bypass graft (Figure 7A). Through endovascular access via the right femoral artery, a dissection stent graft (ZDEG-PT-40-32-205-PF) was implanted into the true lumen of the thoracic aorta, with a coverage of the ostium of the left subclavian artery (Figure 7B). The endovascular access site was surgically closed.

Postoperatively, the patient was evaluated by a cardiologist due to elevated troponin levels. The consultation concluded that the troponin elevation was likely secondary to muscle injury in the context of renal failure or due to perioperative/multiorgan failure-related myocardial injury. The patient did not meet the criteria for myocardial infarction, and there were no indications for coronary angiography or antiplatelet therapy for cardiological reasons. The patient's condition gradually improved day by day, with the normalization of temperature and perfusion in the left lower limb. Motor deficits in the left lower extremity also progressively resolved. The patient was discharged home in stable condition on postoperative day 4.

**Figure 8:**
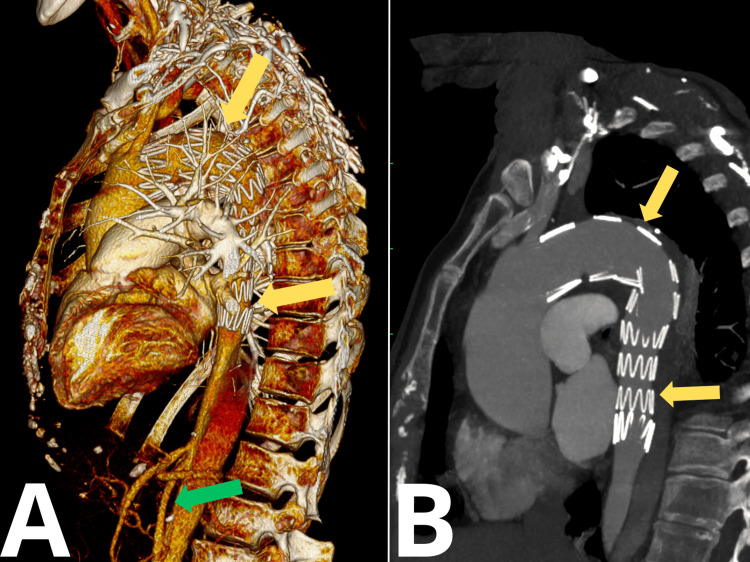
CT During Follow-Up: (A) 3D Reconstruction and (B) Sagittal 2D Plane The yellow arrow indicates the stent graft. The green arrow indicates the patent left common iliac artery CT, computed tomography; 3D, three-dimensional

In June 2024, the patient underwent imaging follow-up consisting of a CT scan with 3D reconstruction (Figure 8). These studies confirmed patent blood flow through the left lower limb and showed no evidence of endoleaks from the stent graft.

During outpatient follow-up several weeks after hospital discharge, the patient reported the gradual development of a painless but pulsatile mass in the right antecubital fossa, corresponding to the site of previous endovascular access. Initially subtle, the lesion progressively enlarged and was associated with a new-onset sensation of fullness and mild warmth in the area. On physical examination, a 4 × 3 cm, soft, compressible mass was palpable in the right antecubital fossa. The lesion exhibited a palpable thrill and an audible bruit on auscultation, raising clinical suspicion for an arteriovenous fistula. The overlying skin was intact without signs of inflammation. The radial and ulnar pulses were present and symmetrical, with no signs of distal ischemia. Capillary refill and neurological function in the right hand were preserved. Although the mass was not painful, its hemodynamic features and the patient's history of arterial puncture at this site supported the clinical diagnosis of an iatrogenic arteriovenous communication.

**Figure 9:**
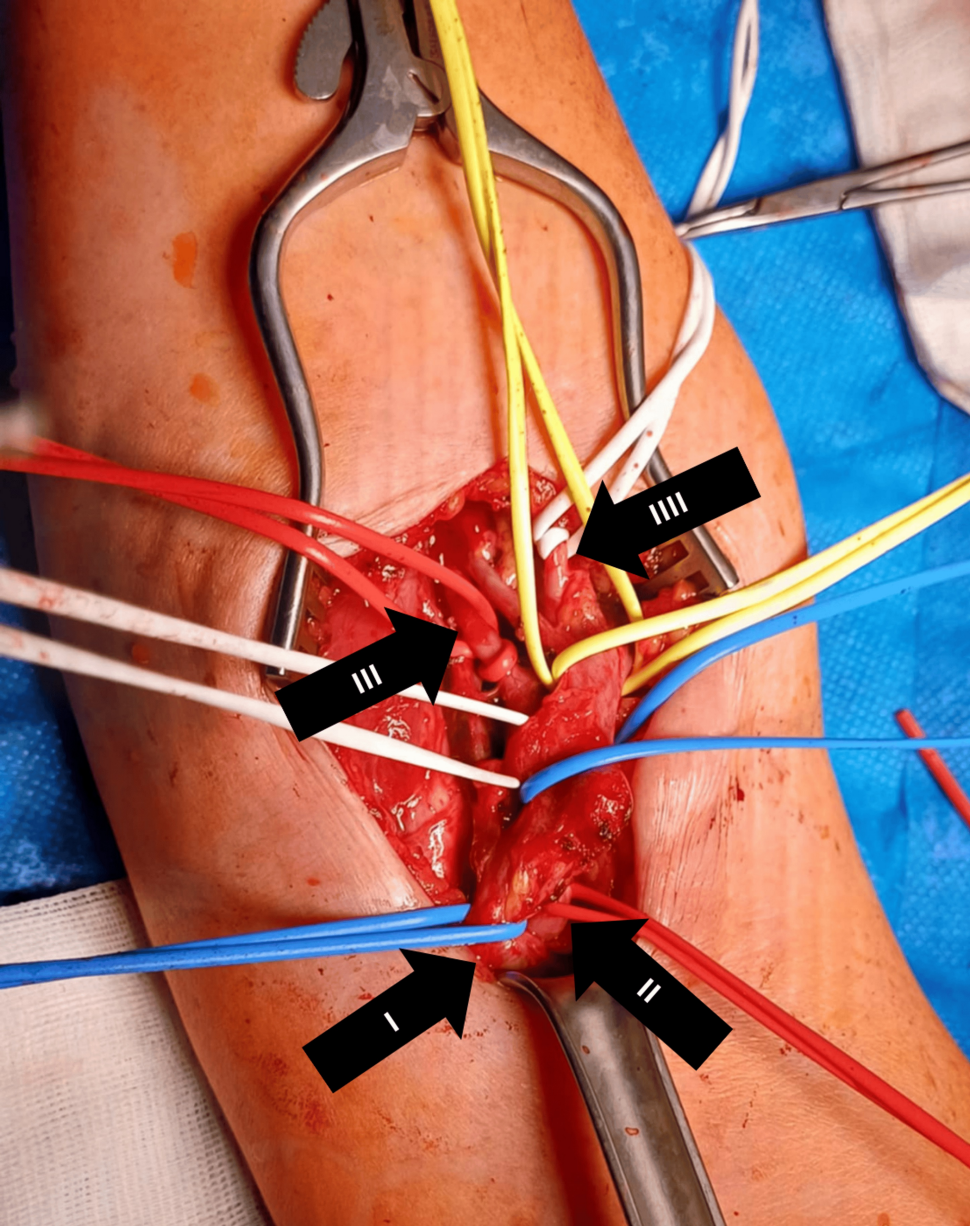
Intraoperative Image of the Right Upper Limb The vessels involved in the arteriovenous fistula have been isolated and looped with vessel loops for identification and control. Upper part of the image, distal part; lower part of the image, proximal part. Arrow I indicates the brachial vein. Arrow II indicates the brachial artery. Arrow III indicates the ulnar artery. Arrow IIII indicates the radial artery

In February 2025, the patient was admitted to our department due to an arteriovenous fistula in the right cubital fossa, following a prior endovascular procedure for aortic dissection. A skin incision was made above the palpable, pulsatile arteriovenous fistula, characterized by an audible bruit, located between the brachial artery and vein. The fistula measured approximately 1 cm in diameter. After the careful dissection of the surrounding arterial and venous structures, due to the risk of hemorrhage, the vessels were isolated with vascular loops (Figure 9). The fistula was then transected, and both ends were closed with 6-0 Prolene (Ethicon, Inc., Raritan, NJ) vascular sutures. Following vessel separation, the normal pulsation of the brachial artery was preserved, and a reduction in the diameter of the brachial vein was noted. Hemostasis was secured, a Redon drain was placed near the fistula site, and the wound was dressed. Postoperatively, palpable pulses were present in the radial and ulnar arteries at the wrist. The first postoperative day was uneventful, and the patient was discharged home.

Approximately two weeks later, during suture removal, clinical follow-up revealed well-palpable pulses over the radial and brachial arteries. There were no symptoms or clinical signs of limb ischemia. The fistula was neither visible nor palpable, and no bruit was detected on auscultation. Unfortunately, the patient most likely experienced a cerebrovascular accident with a fatal outcome, precluding the planned imaging follow-up with Doppler ultrasonography.

## Discussion

This case highlights the diagnostic and therapeutic challenges associated with complicated type B aortic dissection (TBAD) presenting with peripheral malperfusion. While uncomplicated TBAD is typically managed conservatively with strict blood pressure control, the presence of end-organ ischemia, especially critical limb ischemia, necessitates urgent intervention [18]. In our patient, the clinical picture of acute ischemia of the left lower limb with absent distal pulses indicated the dynamic obstruction of the true lumen by the dissection flap, leading to the compromised perfusion of the iliac and femoral arteries. The decision to proceed with an emergency femoro-femoral bypass was guided by the need to restore lower limb perfusion promptly. This approach is supported by reports showing that early revascularization can prevent irreversible ischemic damage and limb loss [19,20]. However, reperfusion in the setting of prolonged ischemia can also trigger systemic inflammatory responses, contributing to multiorgan dysfunction, as seen in this case with acute kidney injury and later gastrointestinal complications [21,22]. Definitive aortic repair with TEVAR was performed as a second-stage procedure. TEVAR has become the preferred treatment modality in complicated TBAD, offering lower perioperative morbidity and mortality compared to open surgery [23]. By covering the proximal entry tear, TEVAR can promote false lumen thrombosis and true lumen re-expansion, alleviating distal malperfusion.

In our patient, the procedure was technically successful; however, the persistent dynamic collapse of the true lumen in the infrarenal segment required adjunctive bare-metal stenting to improve distal perfusion and remodeling. Bare-metal stents commonly used in such cases include the Zenith Dissection Endovascular System (Cook Medical, Bloomington, IN). In this case, we employed this commercially available system and applied the STABILIZE technique, which involves a provisional extension to induce the complete attachment of the stent graft to the aortic wall [24]. Following stent deployment, the ballooning of both the distal stent graft and the bare-metal stent areas was performed to exclude the thoracic false lumen completely and achieve a single-channeled abdominal aorta, thereby optimizing true lumen expansion and perfusion. Despite successful revascularization and endovascular repair, the patient developed late complications, including intestinal ischemia, which may have been multifactorial, related to both initial malperfusion and reperfusion injury [25]. Additionally, the occurrence of an iatrogenic arteriovenous fistula in the right brachial region, likely due to prior arterial access, underscores the importance of careful vascular access management and postprocedural surveillance [26,27]. Although rare, such complications can contribute to long-term morbidity and should be included in follow-up protocols. This case reinforces the need for a multidisciplinary and stepwise approach in managing complicated TBAD, particularly in the presence of malperfusion syndromes. Timely recognition and intervention, supported by imaging and hemodynamic monitoring, are key to optimizing outcomes. Furthermore, it highlights the necessity for prolonged surveillance and the management of delayed complications, both systemic and iatrogenic.

## Conclusions

This case demonstrates the complex and potentially life-threatening course of extensive TBAD complicated by peripheral malperfusion, including critical ischemia of the lower limb. It underscores the importance of rapid diagnosis and immediate intervention, particularly in the presence of acute limb ischemia, which poses a direct threat to limb viability and overall patient prognosis. The staged therapeutic approach, initial surgical revascularization via femoro-femoral bypass to restore distal perfusion, followed by definitive endovascular aortic repair, proved to be an effective strategy for stabilizing the dissection and preventing further ischemic damage. However, despite successful revascularization and stent graft placement, the patient developed serious systemic complications, including acute kidney injury and intestinal ischemia, illustrating the broader physiological consequences of aortic malperfusion and reperfusion injury. Furthermore, the delayed presentation of an iatrogenic brachial arteriovenous fistula highlights the need for long-term vigilance after endovascular procedures, as vascular access-related complications may not manifest immediately. Overall, this case emphasizes the need for a multidisciplinary approach involving vascular surgery, interventional radiology, and critical care teams to ensure timely diagnosis, tailored intervention, and comprehensive postoperative monitoring. The effective management of such complex aortic presentations requires not only technical expertise in hybrid surgical-endovascular procedures but also awareness of potential systemic and iatrogenic complications that can significantly influence outcomes.
